# New reassortant H5N8 highly pathogenic avian influenza virus from waterfowl in Southern China

**DOI:** 10.3389/fmicb.2015.01170

**Published:** 2015-10-23

**Authors:** Yafen Song, Jin Cui, Hui Song, Jiaqi Ye, Zhishan Zhao, Siyu Wu, Chenggang Xu, Peirong Jiao, Ming Liao

**Affiliations:** ^1^National and Regional Joint Engineering Laboratory for Medicament of Zoonosis Prevention and ControlGuangzhou, China; ^2^Key Laboratory of Animal Vaccine Development, Ministry of AgricultureGuangzhou, China; ^3^Key Laboratory of Zoonosis Prevention and Control of GuangdongGuangzhou, China; ^4^College of Veterinary Medicine, South China Agricultural UniversityGuangzhou, China; ^5^College of Life Science, South China Agricultural UniversityGuangzhou, China

**Keywords:** H5N8, highly pathogenic avian influenza virus, reassortant, waterfowl, Southern China

## Abstract

New reassortant H5N8 highly pathogenic avian influenza viruses were isolated from waterfowl in Southern China. Blast analysis demonstrated that the PB2 gene in these viruses were most closely related to A/wild duck/Shangdong/628/2011 (H5N1), while their NP genes were both more closely related to A/wild duck/Shandong/1/2011 (H5N1) and A/duck/Jiangsu/k1203/2010 (H5N8). However, the HA, NA, PB1, PA, M, and NS genes had the highest identity with A/duck/Jiangsu/k1203/2010 (H5N8). Phylogenetic analysis revealed that their HA genes belonged to the same GsGd H5 clade 2.3.4.4 detected in China in 2010. Therefore, we supposed that these H5N8 viruses might be novel reassortant viruses that have a H5N8 backbone while acquiring PB2 and NP genes from H5N1 viruses. This study is useful for better understanding the genetic and antigenic evolution of H5 avian influenza viruses in Southern China.

## Introduction

Aquatic birds, including wild and domestic waterfowl, are the original reservoir of influenza A viruses, which provide the genetic diversity of influenza viruses and contribute to create new human and bird pandemic influenza viruses (Yoon et al., 2014). About three out of five of the world’s population of domestic ducks (about 600 million ducks) is raised in Southern China, which intimates the largest waterfowl reservoir for influenza viruses on the earth (Huang et al., 2010). Domestic ducks and geese are regarded as intermediate agencies between the aquatic bird and terrestrial poultry in the influenza virus ecosystem (Huang et al., 2010).

In 1996, the H5N1 highly pathogenic avian influenza virus (HPAIV) (A/goose/Guangdong/1/1996) was first isolated from sick geese during an outbreak in Guangdong of China (Xu et al., 1999). In 1997, the GSGD/96-like H5N1 avian influenza virus (AIV) caused eighteen infected patients and six dead persons in Hong Kong of China, which was first reported that H5N1 AIV transmitted from birds to humans (Claas et al., 1998; Subbarao et al., 1998; Xu et al., 1999). To date, H5N1 HPAIV has caused 840 human cases worldwide, including 447 deaths (WHO, 2015). In 1961, an H5 highly pathogenic AIV, A/Tern/South Africa/61, was first isolated from shorebirds (Xu et al., 1999). In recent years, various subtypes of HPAIVs bearing H5 HA (H5N2, H5N3, H5N5, H5N6, and H5N8) have been detected in wild and domestic birds (like ducks, geese, quails, and chickens) and even in humans Especially in 2014, H5N8 viruses could be isolated from nine countries and different birds. So far, no human cases related to H5N8 viruses have been reported anywhere. However, in recent reports, it has been demonstrated that mammals such as mice, ferrets, dogs, and cats could be infected by H5N8 viruses showing mild clinical disease (Kim et al., 2014). Some research had revealed that HPAIV, whose HA gene originate from clade 2.3.4.4 in the wild and domestic waterfowl were able to bind to both the avian receptor and human receptor (Kim et al., 2014; Li et al., 2014). If these H5N8 viruses spread like avian influenza A (H5N1) viruses, they could cause numerous outbreaks in poultry and pose a serious threat to human health.

In our study, two new H5N8 viruses were isolated from waterfowl in Southern China between 2013 and 2014. We sequenced the entire viral genome and performed the phylogenetic analysis and determined the molecular characteristics of these viruses.

## Materials and Methods

### Viruses

During our active surveillance, the H5N8 AIVs, A/goose/Guangdong/s13124/2013 (H5N8) (GDs13124) and A/duck/Guangdong/s14044/2014 (H5N8) (GDs14044), were isolated from fecal samples of healthy white ducks and black mane geese in live bird market of Guangdong, Southern China between 2013 and 2014. These H5N8 viruses were identified by reverse-transcription polymerase-chain reaction (RT-PCR), hemagglutination test, and hemagglutination inhibition (HI) test as per the standard protocol (WHO, 2002; Nagarajan et al., 2009; Office International des Epizooties (OIE), 2015). Moreover, these isolates were identified again as H5N8 viruses by nucleotide sequence and a BLAST search of the Influenza Sequence Datebase in GeneBank. Briefly, fecal samples were inoculated into the allantoic cavity of 9 to 10-day-old specific pathogen free (SPF) embryonated chickens eggs. The allantoic fluid was harvested after incubation at 37°C for 48 h. The HI test was done using four HA units of the isolates and subtype specific anti-sera obtained from Harbin Veterinary Research Institute, China. Viral RNA was extracted from allantoic fluid using Trizol LS Reagent (Life Technologies, Inc.) and transcribed into cDNA with using universal 12-mer Uni12 primer AGCAAAAGCAGG (Hoffmann et al., 2001) and Superscript III reverse transcriptase (Invitrogen, China). PCR was performed using primers as described by Hoffmann et al. (2001) and Jiao et al. (2008). The PCR program included an initial denaturation at 95°C for 5 min; 35 cycles of denaturation at 94°C for 1 min, annealing at 53°C for 1 min and extension at 72°C for 2 min 30 s; and a final elongation step at 72°C for 10 min. After the subtype was confirmed, the viruses were subsequently passaged three times with the inoculation of 9 to 10-day-old SPF embryonated chickens eggs by limiting dilution assay. At the same time, subtype assay was confirmed again. Finally, virus allantoic fluid were harvested and stored at –80°C before use. Values of 50% egg infective doses (EID_50_) and 50% egg lethal doses (ELD_50_) were calculated using the Reed–Muench method (Thakur and Fezio, 1981). All experiments with H5 subtype AIVs were carried out in BSL-3 conditions.

### Sequence Analysis

The eight segments of the two viruses were generated using primers as described above. The PCR procedure was alike as described above. The PCR products were purified with the QIAquick PCR purification kit (QIAGEN) following the manufacturer’s instructions and sequencing was performed by using an ABI Prism 3730 genetic analyser (Applied Biosystems) by Shanghai Invitrogen Biotechnology Co., Ltd. All sequences were assembled, edited, alignment, and residue analysis using Lasergene 7.1 (DNASTAR) (Yuan et al., 2014). Neighbor-joining trees of these H5N8 viruses were created by MEGA 4.0 (Sinauer Associates, Inc., Sunderland, MA, USA). The reliability of the phylogenies was estimated by performing 1000 bootstrap replicates (Smith et al., 2006; Zhao et al., 2013; Jeong et al., 2014; WHO/OIE/FAO H5N1 Evolution Working Group, 2014). The sequences of our isolates can be obtained from GenBank under the accession numbers (KT383475-KT383490).

## Results

GDs13124 and GDs14044 grew efficiently in eggs with virus titres of 8.83 and 8.38 log_10_ 50% egg infectious dose (EID_50_)/0.1 mL, respectively. The viruses were highly pathogenic (HP) in eggs with titres of 6.75 and 7.50 log_10_50% egg lethal dose (ELD_50_)/0.1 mL, respectively.

To understand the genetic properties of these viruses, all eight genes of GDs13124 and GDs14044 were characterized and phylogenetically analyzed, respectively. Our sequence data were compared with the reference sequence from the NCBI Influenza Virus Resource.

**FIGURE 1:**
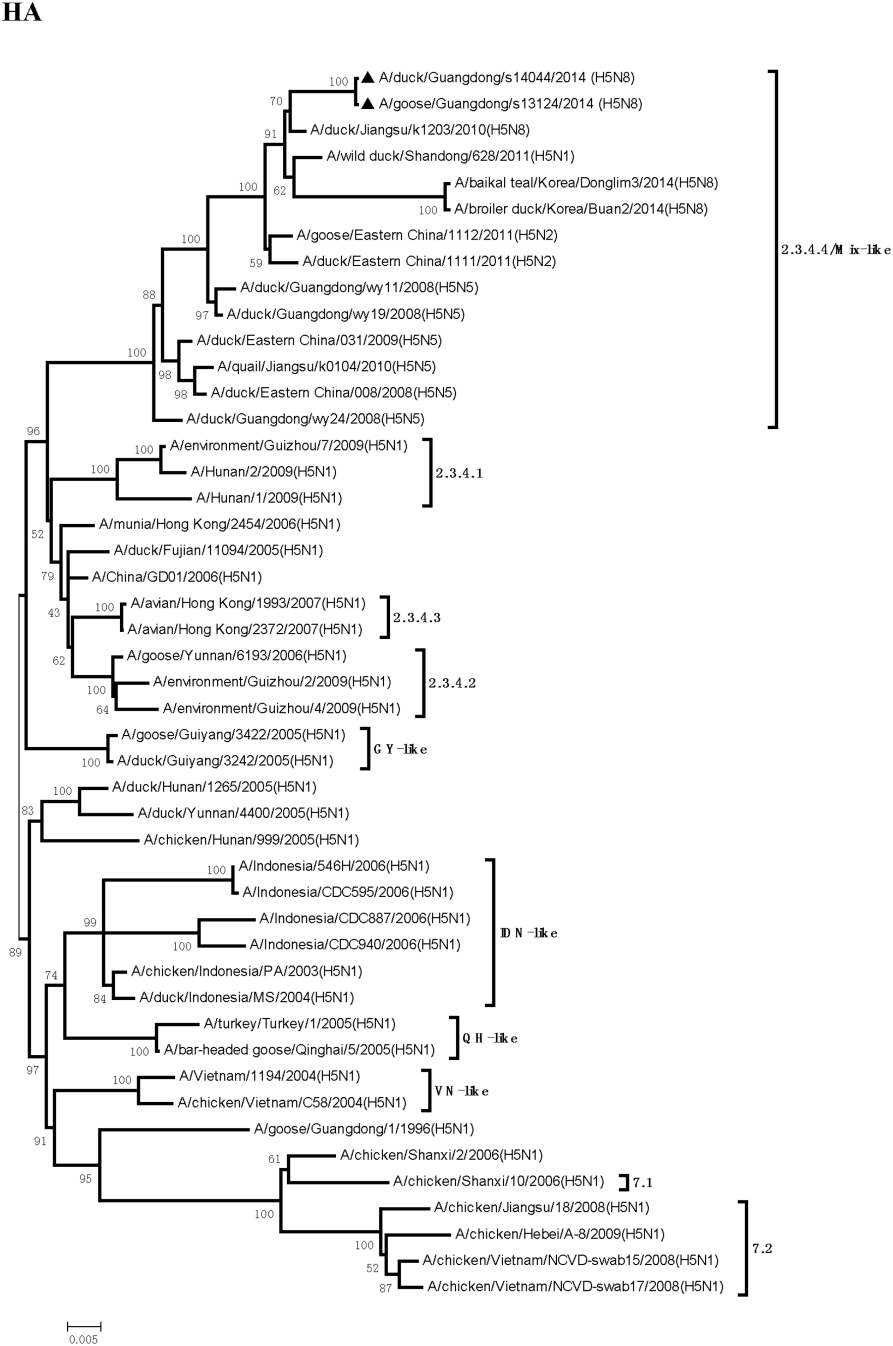
**Phylogenetic analysis of hemagglutinin (HA).** The trees were constructed by using the neighbor joining method with the Maximum Composite Likelihood model and MEGA version 4.0 (http://www.megasoftware.net) with 1,000 bootstrap replicates based on the following sequences: HA (A): nucleotides (nt) 1 to 1704. IDN, Indonesia; QH, Qinghai; VN, Vietnam; GY, Guiyang.

**FIGURE 2:**
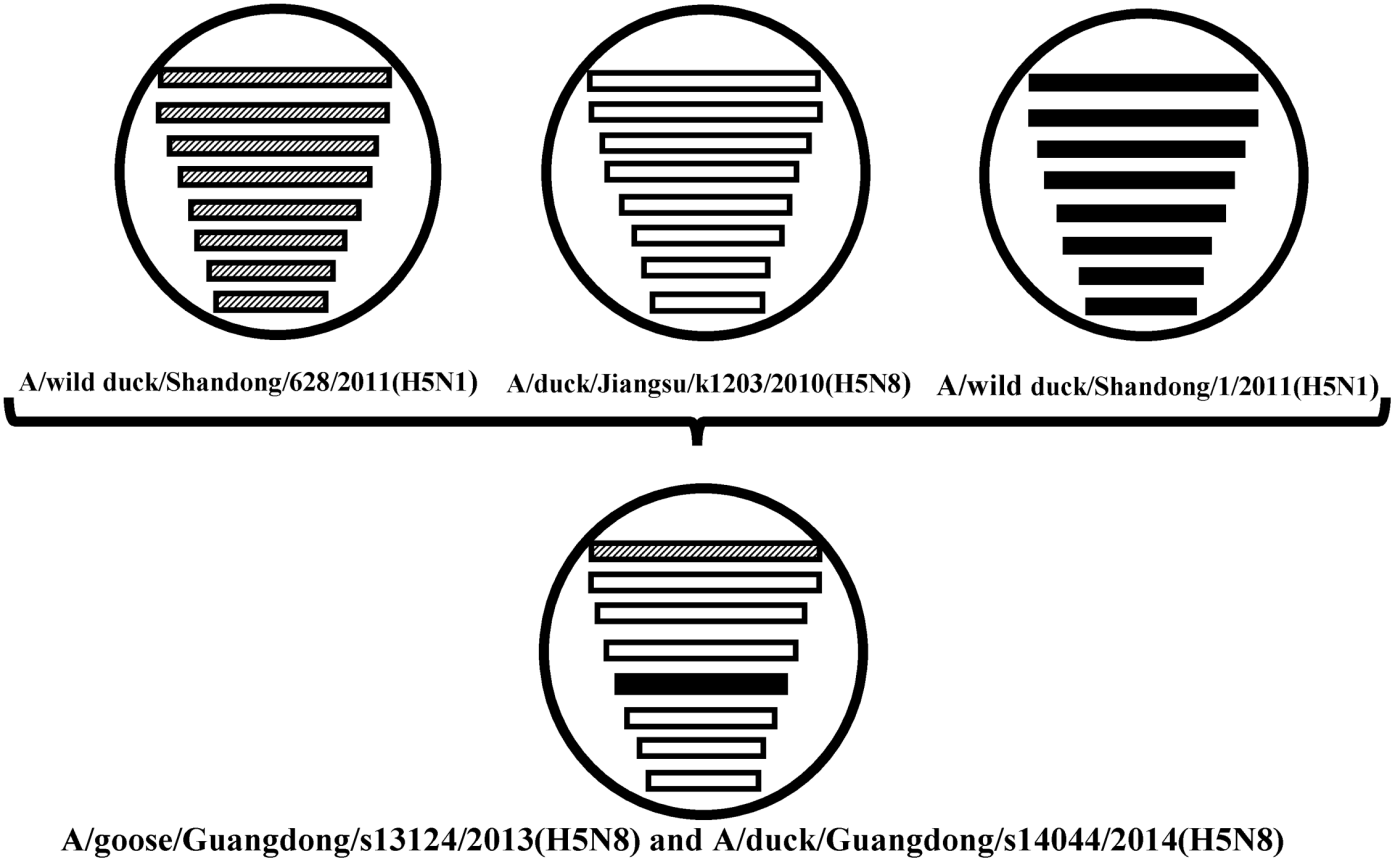
**The eight gene segments of two novel H5N8 viruses, represented by horizontal bars are, from top to bottom, polymerase basic subunit 2 (PB2), polymerase basic subunit 1 (PB1), polymerase acidic subunit (PA), hemagglutinin (HA), nucleoprotein (NP), neuraminidase (NA), matrix (M), and non-structural (NS).** Each different color represents a distinct origin.

Sequence analysis showed that all eight genes of GDs13124 and GDs14044 had 99.4 to 100% identity. The HA gene of both the viruses belonged to Mix-like sublineage and were clustered into the clade 2.3.4.4 (**Figure 1**) (Gu et al., 2013). The HA gene of GDs13124 and GDs14044 were both more closely related to the A/duck/Jiangsu/k1203/2010 (H5N8) virus circulating in birds in Eastern China around 2009–2010 (Gu et al., 2013). As shown in **Tables 1** and **2** and **Figure 2**, the nucleotide identities among these were up to 99.8%. The viruses were highly pathogenic with the multi-basic cleavage site (LREKRRKR↓GL) in the HA molecule. The HA gene of both encoded 567 amino acids. 182, 222, and 224 amino acid sites were still Asn, Gln, Gly in the receptor binding pocket of the HA1 (H5 numbering used throughout), respectively, suggesting that the two viruses preferentially bind to the avian-like NeuAca2,3-Gal receptor rather than the human-like NeuAca2,6-Gal receptor (Ha et al., 2001; Yamada et al., 2006).

**Table 1:** Avian influenza viruses (AIVs) with highest nucleotide sequence homology to H5N8 AIV (A/goose/Guangdong/s13124/2013) as determined by BLAST search in the GenBank^a^.

Gene^b^	Virus with the highest percentage of nucleotide homology	Homology (%)
PB2	A/wild/duck/Shandong/628/2011(H5N1)	98.6
PB1	A/duck/Jiangsu/k1203/2010(H5N8)	98.9
PA	A/duck/Jiangsu/k1203/2010(H5N8)	99.2
HA	A/duck/Jiangsu/k1203/2010(H5N8)	98.8
NP	A/wild duck/Shandong/1/2011(H5N1)	98.4
	A/duck/Jiangsu/k1203/2010(H5N8)	98.4
NA	A/duck/Jiangsu/k1203/2010(H5N8)	97.6
M	A/duck/Jiangsu/k1203/2010(H5N8)	99.2
NS	A/duck/Jiangsu/k1203/2010(H5N8)	98.9

*^a^http://www.ncbi.nlm.nih.gov/.*

*^b^HA, hemagglutinin; NA, neuraminidase; PB, polymerase basic subunit; PA, polymerase acidic subunit; NP, nucleoprotein; M, matrix; NS, non-structural.*

**Table 2:** Avian influenza viruses with highest nucleotide sequence homology to H5N8 AIV (A/duck/Guangdong/s14044/2014) as determined by BLAST search in the GenBank^a^.

Gene^b^	Virus with the highest percentage of nucleotide homology	Homology (%)
PB2	A/wild/duck/Shandong/628/2011(H5N1)	98.6
PB1	A/duck/Jiangsu/k1203/2010(H5N8)	98.9
PA	A/duck/Jiangsu/k1203/2010(H5N8)	99.2
HA	A/duck/Jiangsu/k1203/2010(H5N8)	98.8
NP	A/wild duck/Shandong/1/2011(H5N1)	98.4
	A/duck/Jiangsu/k1203/2010(H5N8)	98.4
NA	A/duck/Jiangsu/k1203/2010(H5N8)	97.7
M	A/duck/Jiangsu/k1203/2010(H5N8)	99.2
NS	A/duck/Jiangsu/k1203/2010(H5N8)	98.9

*^a^http://www.ncbi.nlm.nih.gov/.*

*^b^HA, hemagglutinin; NA, neuraminidase; PB, polymerase basic subunit; PA, polymerase acidic subunit; NP, nucleoprotein; M, matrix; NS, non-structural.*

**FIGURE 3:**
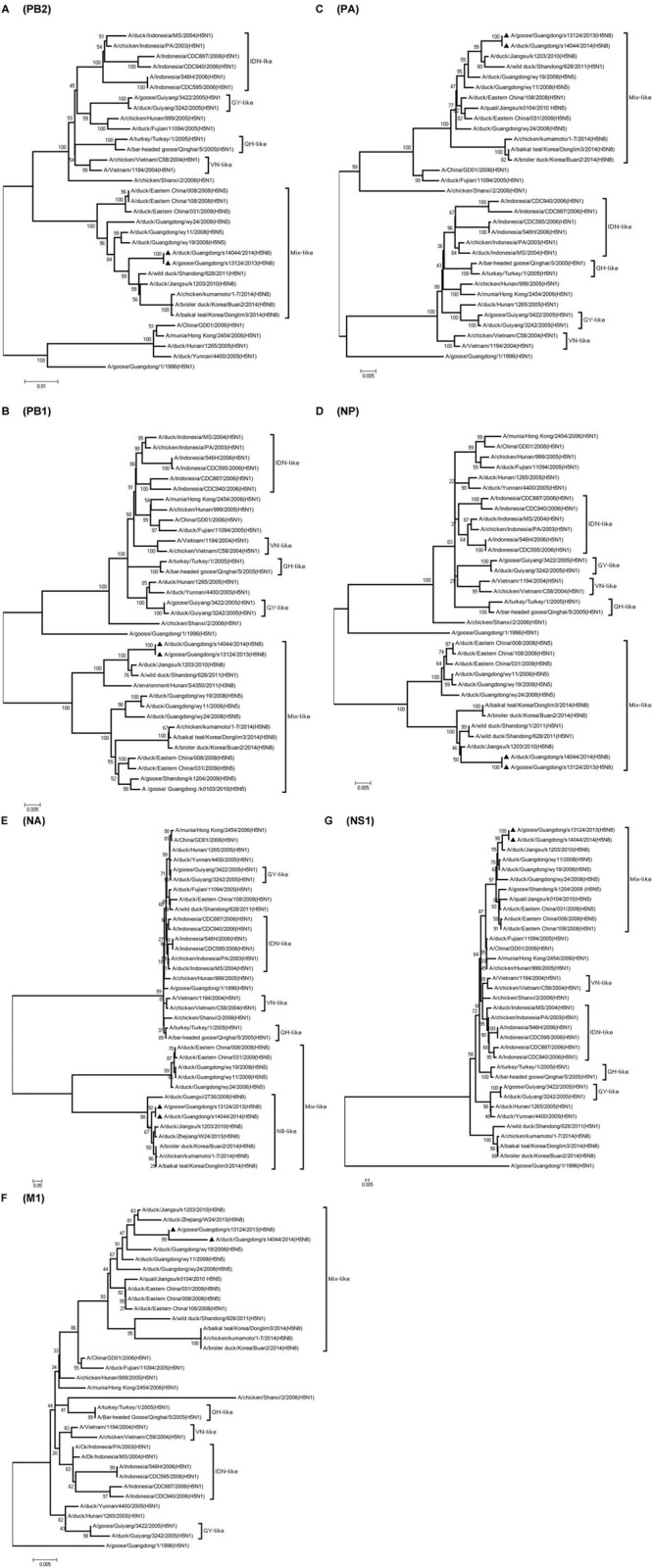
**Phylogenetic analysis of PB2, PB1, PA, NP,NA, M, NS.** The trees were constructed by using the neighbor joining method with the Maximum Composite Likelihood model and MEGA version 4.0 (http://www.megasoftware.net) with 1,000 bootstrap replicates based on the following sequences: PB2 **(A)**: nt 28 to 2307; PB1 **(B)**: nt 25 to 2298; PA **(C)**: nt 25 to 2175; NP **(D)**: nt46 to 1542; NA **(E)**: nt 1 to 1413; M1 **(F)**: nt 26 to 784; NS1 **(G)**: nt 27 to 704. IDN, Indonesia; QH, Qinghai; VN, Vietnam; GY, Guiyang.

Phylogenetic analyses showed that the NA gene of both viruses fell into the N8-like sublineage and that the viruses had high identity with each other (**Figure 3E**). As shown in **Tables 1** and **2** and **Figure 2**, the NA genes were closest to A/duck/Jiangsu/k1203/2010 (H5N8), with a nucleotide identity of 97.6 and 97.7%, respectively. No stalk deletion was found in the NA gene. Both viruses had 96-Ala and 258-Gln in the NA gene, indicating that they may reduce susceptibility to oseltamivir or zanarnivir (Zhong et al., 2014).

The PB2 and NP genes were also clustered into the Mix-like sublineage (**Figures 3A,D**). The PB2 gene of both viruses had 98.6% identity compared with the A/wild duck/Shangdong/628/2011 (H5N1). The NP genes were both more closely related to A/wild duck/Shandong/1/2011 (H5N1) and A/duck/Jiangsu/k1203/2010 (H5N8), with a nucleotide identity of 98.4%, respectively (**Tables 1** and **2**; **Figure 2**). The PB2 is an important factor related to the host range and virulence of influenza viruses. E627K or D701N substitutions were thought to contribute to the adaptation, replication, and virulence of H5N1 viruses in humans and mice (Li et al., 2005; Le et al., 2009; Liu et al., 2010; Gu et al., 2013). The 627 and 701 amino acid residues were still E and D in the GDs13124 and GDs14044 virus, respectively. The PB1, PA, M, and NS genes were derived from the Mix-like sublineage, respectively (**Figures 3B,C,F,G**). As shown in **Tables 1** and **2** and **Figure 2**, the PB1, PA, and NS genes had the highest identities with A/duck/Jiangsu/k1203/2010 (H5N8) (PB1 98.9%, PA 99.2%, and NS 98.9%). The M genes were both closest to the A/duck/Jiangsu/k1203/2010 (H5N8) virus with nucleotide identities of 99.2 and 98.6%, respectively. There was an S31N mutation in the M2 protein indicating that these H5N8 viruses may reduce susceptibility to adamantanes and rimantadine (Cheung et al., 2006). The substitution at position S42, F98, and M101 of the NS1 protein could increase virulence in mice (Guan et al., 1999). Both of the NS1 genes of these H5N8 viruses possess those amino acids, suggesting that they may increase virulence in mice. The mutation at position S200 in the NS1 gene may decrease antiviral response in hosts when coupled with A47 in the NS2 gene (Imai et al., 2010), both of which were found in these H5N8 viruses.

## Discussion

The H5N8 AIVs have been previously found in Asia, Europe, and North America. In 2014, the viruses emerged again in Japan, Korea, Northeast China, Germany, Italy, Netherlands, Russia, the United Kingdom, and the USA. So, the H5N8 viruses gradually spread in many countries, cause enormous economic losses to poultry industry and may have a threat to human healthy. We first isolated the H5N8 viruses (GDs13124 and GDs14044) from waterfowl in Southern China between 2013 and 2014. Our results demonstrated that the PB2 gene of these viruses had the highest identity with A/wild duck/Shangdong/628/2011 (H5N1); their NP genes were both more closed to A/wild duck/Shandong/1/2011 (H5N1) and A/duck/Jiangsu/k1203/2010 (H5N8); the other genes were more close to A/duck/Jiangsu/k1203/2010 (H5N8). The HA gene of these viruses were both clustered into clade 2.3.4.4 (Gu et al., 2013). The NA gene of these viruses belonged to the N8-like lineage and the other genes all belonged to Mix-like lineage, so the genes might originate from a new gene pool that includes multiple subtypes. The new Mix-like gene pool characterized by the H5N8 and H5N5 subtype AIVs from Eastern China also includes a small number of H5N1 and H5N2 viruses. Specially, a notable case concerning the H5N1 gene pool has widened and more and more new branches have emerged since 2003 (WHO/OIE/FAO H5N1 Evolution Working Group, 2014). In the future, the new Mix-like gene pool may become wider and more complicated like the H5N1 gene pool, even generating more new branches. Therefore, we must do more virological investigation and surveillance for AIVs to monitor the change of the new Mix-like gene pool. Additionally, human infection with the H5N6 viruses belonging to this new Mix-like gene pool was first reported in the Sichuan province of China in May 2014 and then later in Guangdong in December 2014. So the viruses in the new Mix-like gene pool are a threat to human health in China.

Even though massive slaughter of poultry and vaccination strategies were used to prevent and control the H5 HPAI viruses in China, the H5 HPAI viruses still circulated continuously in waterfowl in Southern China and frequently provided gene segments to generate new strains (Wei et al., 2014). Between 2004 and 2009, multiple clades (2.2, 2.5, 2.3.1, 2.3.2, 2.3.3, 2.3.4, 7, 8, and 9) of H5N1 HPAI were identified by surveillance in China (Guan et al., 1999; Liu et al., 2010; WHO/OIE/FAO H5N1 Evolution Working Group, 2014). Clade 2.3.4 predominantly circulated in poultry in South China from 2005 to 2012. Furthermore, various NA subtypes of H5 viruses (H5N2, H5N5, and H5N8) containing the gene of clade 2.3.4 (H5N1) viruses have been found in ducks, geese, quails, and chickens (Liu et al., 2010; Zhao et al., 2012, 2013). To protect poultry against H5N1 influenza viruses in clade 2.3.4, the Re-5 inactivated vaccine, whose HA and NA genes originated from A/duck/Anhui/1/2006 (clade 2.3.4), were widely used around China until June 2012 (Liu et al., 2010). In our study, the HA gene of the GDs13124 and GDs14044 viruses belonged to clade 2.3.4.4. These results suggested that the clade 2.3.4 viruses was still circulating in China and generated more subclade, such as 2.3.4.1, 2.3.4.2, 2.3.4.3, 2.3.4.4 (WHO/OIE/FAO H5N1 Evolution Working Group, 2014; WHO, 2015).

In summary, our results showed that the new H5N8 HPAI viruses may come from reassortant between the A/duck/Jiangsu/k1203/2010 (H5N8) virus and other H5N1 viruses, and their HA genes belonged to the same GsGd H5 clade 2.3.4.4. Therefore, this study is useful for better understanding the genetic and antigenic evolution of H5 AIVs in Southern China.

## Conflict of Interest Statement

The authors declare that the research was conducted in the absence of any commercial or financial relationships that could be construed as a potential conflict of interest.
